# MicroRNA expression patterns and signalling pathways in the development and progression of childhood solid tumours

**DOI:** 10.1186/s12943-017-0584-0

**Published:** 2017-01-19

**Authors:** Anna L. Leichter, Michael J. Sullivan, Michael R. Eccles, Aniruddha Chatterjee

**Affiliations:** 1 Department of Pathology, Dunedin School of Medicine, University of Otago, 56 Hanover Street, P.O. Box 913, Dunedin, 9016 New Zealand; 2 Maurice Wilkins Centre for Molecular Biodiscovery, Level 2, 3A Symonds Street, Auckland, New Zealand; 3 Royal Children’s Hospital, Melbourne, VIC Australia

**Keywords:** MicroRNAs, Early development, Childhood solid tumours, Neuroblastoma, Medulloblastoma, Osteosarcoma, Wilms Tumour, Hepatoblastoma

## Abstract

The development of childhood solid tumours is tied to early developmental processes. These tumours may be complex and heterogeneous, and elucidating the aberrant mechanisms that alter the early embryonic environment and lead to disease is essential to our understanding of how these tumours function. MicroRNAs (miRNAs) are vital regulators of gene expression at all stages of development, and their crosstalk via developmental signalling pathways is essential for orchestrating regulatory control in processes such as proliferation, differentiation and apoptosis of cells. Oncogenesis, from aberrant miRNA expression, can occur through amplification and overexpression of oncogenic miRNAs (oncomiRs), genetic loss of tumour suppressor miRNAs, and global miRNA reduction from genetic and epigenetic alterations in the components regulating miRNA biogenesis. While few driver mutations have been identified in many of these types of tumours, abnormal miRNA expression has been found in a number of childhood solid tumours compared to normal tissue. An exploration of the network of key developmental pathways and interacting miRNAs may provide insight into the development of childhood solid malignancies and how key regulators are affected. Here we present a comprehensive introduction to the roles and implications of miRNAs in normal early development and childhood solid tumours, highlighting several tumours in depth, including embryonal brain tumours, neuroblastoma, osteosarcoma, Wilms tumour, and hepatoblastoma. In light of recent literature describing newer classifications and subtyping of tumours based on miRNA profiling, we discuss commonly identified miRNAs, clusters or families associated with several solid tumours and future directions for improving therapeutic approaches.

## Background

MicroRNAs (miRNAs) are a group of small non-coding RNAs, around 19–22 nucleotides in length, first discovered in nematodes (*Caenorhabditis elgans*) in 1993 [1]. Now there are thousands of miRNAs identified in many different eukaryote species [2]. Previously, miRNAs and other noncoding RNAs were dismissed as transcriptional noise [3, 4]. However, recently the ENCODE project revealed that in the human genome there is a vast network of important interactions that occur between regulatory elements, primarily composed of transcription factors and miRNAs [5–7]. miRNAs can regulate gene expression in several ways, through DNA interaction, translational repression or by directly targeting mRNA degradation, and they play vital roles in nearly all biological pathways [8]. With this characterization we now know different sets of miRNAs are expressed in specific cell and tissue types and therefore they play an important role in shaping cellular identity [9, 10]. In addition, miRNAs have been shown to regulate oncogenes, tumour suppressor genes and have an impact on cell cycle control, apoptosis, cell migration and angiogenesis [11–19]. Taken together, these discoveries provide insight into the importance of the roles of miRNAs in maintaining normal cellular function and how disruptions in miRNA expression profiles may heavily impact the development, differentiation, and control of growth leading to diseases such as cancer.

Cancer is a disease of diverse genetic and epigenetic complexity involving changes in gene expression [20–22]. For a long time, carcinogenesis was largely attributed to abnormalities in oncogenes and tumour suppressing genes. It is now widely recognized that miRNAs also play critical roles in cancer. Aberrant miRNA expression associated with cancer development can occur through a number of different types of mechanisms, each of which is a critical event that could greatly alter early development and give rise to the pathogenesis of childhood cancers.

There has been recent work indicating that global miRNA profiles are able to classify human cancers tissues, including paediatric solid tumours [23, 24]. An investigation into biomarkers and risk stratification in childhood solid tumours highlighted that specific serum miRNA profiles can be identified in childhood solid tumours. The results supported the proposed use of miRNA profiling from serum as a non-invasive method with the potential to diagnose childhood solid tumours [25]. Also of note is the emerging evidence of disrupted components of the miRNA biogenesis pathway, which, if altered, affect miRNA expression levels on a global scale. Enzymes involved in the production of mature miRNAs have been implicated in certain childhood cancers, including neuroblastomas and Wilms tumour [26–28].

In recent years, the concepts of cell of origin (COO) and cancer stem cells (CSCs) have gained traction; these concepts are distinct. The COO is a normal cell, which has acquired the first cancer promoting mutation, and CSCs are the cellular component of a tumour that maintains malignant growth. Therefore, COOs and CSCs are cancer initiating and cancer propagating respectively. Investigators have identified a number of miRNAs associated with CSCs, differentially expressed in six paediatric solid tumour cell lines [29]. The identified potential targets were further investigated using *in silico* biological analytical processes, which revealed four annotated pathways that were enriched: cell cycle, cell proliferation, p53 and TGF-beta/BMP. This highlights an important consideration; the actions of miRNAs on key critical developmental pathways could have major effects on how these pathways function, and could be deleteriously affected by deregulated miRNAs, causing a chain of events which may lead to an environment where cancer develops [30].

The scope of this review aims to address the current state of miRNA research in relation to childhood solid tumours. Due to the necessarily precise genetic regulation that occurs during development, it is plausible that miRNAs play a major role in childhood cancers. A deeper understanding of the links between the deregulation of miRNAs and childhood cancers can improve the way children are diagnosed and treated. Here we briefly describe miRNA biogenesis, how miRNAs function in early development and their ability to act as tumour suppressors and oncomiRs. In addition, the key developmental pathways and how they lead to malignancies in different organs is examined through the lens of miRNA action. We explore several childhood solid tumours in detail, neuroblastoma, osteosarcoma, nephroblastoma (Wilms tumour), hepatoblastoma, and several types of brain tumours. In the following discussion of these solid tumour types, miRNAs (or families) identified as aberrantly expressed in multiple tumour types are highlighted in an effort to understand where connections between the developmental processes and childhood solid tumours might be used as a basis for discovering shared mechanisms. It remains challenging to interpret how the many targets of individual miRNAs directly affect downstream processes, leading to development or progression of childhood cancer; however, pending accurate interpretations, this may lead to the discovery of targetable pathways and assist in the generation of new therapies.

### The miRNA biogenesis process

miRNAs are initially processed from precursor molecules (pri-miRNAs) in the nucleus [31, 32]. Pri-miRNAs are transcribed by RNA polymerase II/III from independent genes, or intronic sequences. The pri-miRNAs fold into hairpins, and are cleaved by the enzymes Drosha and Dicer in a two-step processing function [33, 34]. First the nuclear localised complex of DROSHA and DGCR8 cleaves the pri-miRNA, producing a ~70 nucleotide pre-miRNA. This is exported to the cytoplasm by the complex Exportin 5-Ran-GTP, where TRBP and DICER process the pre-miRNA to yield a ~20 bp miRNA/miRNA* coupled pair [35, 36]. One strand represents the 5’ miRNA, whereas the other strand represents the 3’ miRNA. Depending on the specific miRNA one of the strands is preferentially degraded over the other more highly expressed miRNA [37]. The preferentially expressed miRNA is incorporated into the miRNA induced silencing complex (miRISC) which allows miRNAs to target mRNAs and induce translational repression or deadenylation, and subsequent degradation, leading to rapid silencing of the targeted mRNA transcript gene product [38–41].

### miRNA mediated regulation of the genome and key developmental pathways

Through evolution, miRNAs and their mechanistic features for targeting mRNA have been strongly conserved. An important class of gene regulatory molecules, they are often considered “master regulators” of gene expression, because a single miRNA can bind to, and consequently impact the expression of a number of different transcripts. Previous evidence has suggested that the average miRNA has approximately 100 target sites [42]. At the current time miRTarBase, (an experimentally validated miRNA target interaction database), states there are 2,619 known human miRNAs validated to target 12,738 genes [43].

The reason miRNAs can affect multiple targets is due to the mechanism of action in which the miRNAs base-pair to their targets, involving flexible complementary pairing with the mRNA. miRNAs base-pair to their target mRNA on the 3’-UTR, where there are miRNA recognition elements (MREs). miRNAs implement the use of a critical region referred to as the “seed region”, a region on the 5’ end of the miRNA containing 2–8 nucleotides [44, 45]. Interestingly, asymmetry is commonly observed for matches between the miRNA and its target. The 5’ end of miRNA tends to have a greater number of complementary bases than the 3’ end. A key finding indicates that as few as seven base pairs of complementarity to the miRNA 5’ end are sufficient to provide regulation [46]. Additionally, 3’ end complementarity may serve as a specificity factor, allowing the possibility of different sites to become differentially regulated by different miRNA family members. These attributes are necessary in development, where dynamic protein expression occurs in order to orchestrate a number of critical actions, such as cell lineage decisions and the resulting morphogenetic events. If additionally we consider that there are a surprisingly large number of miRNA target sites within the genome, it becomes apparent that miRNAs are capable of a widespread impact on gene regulation [42].

**Fig. 1:**
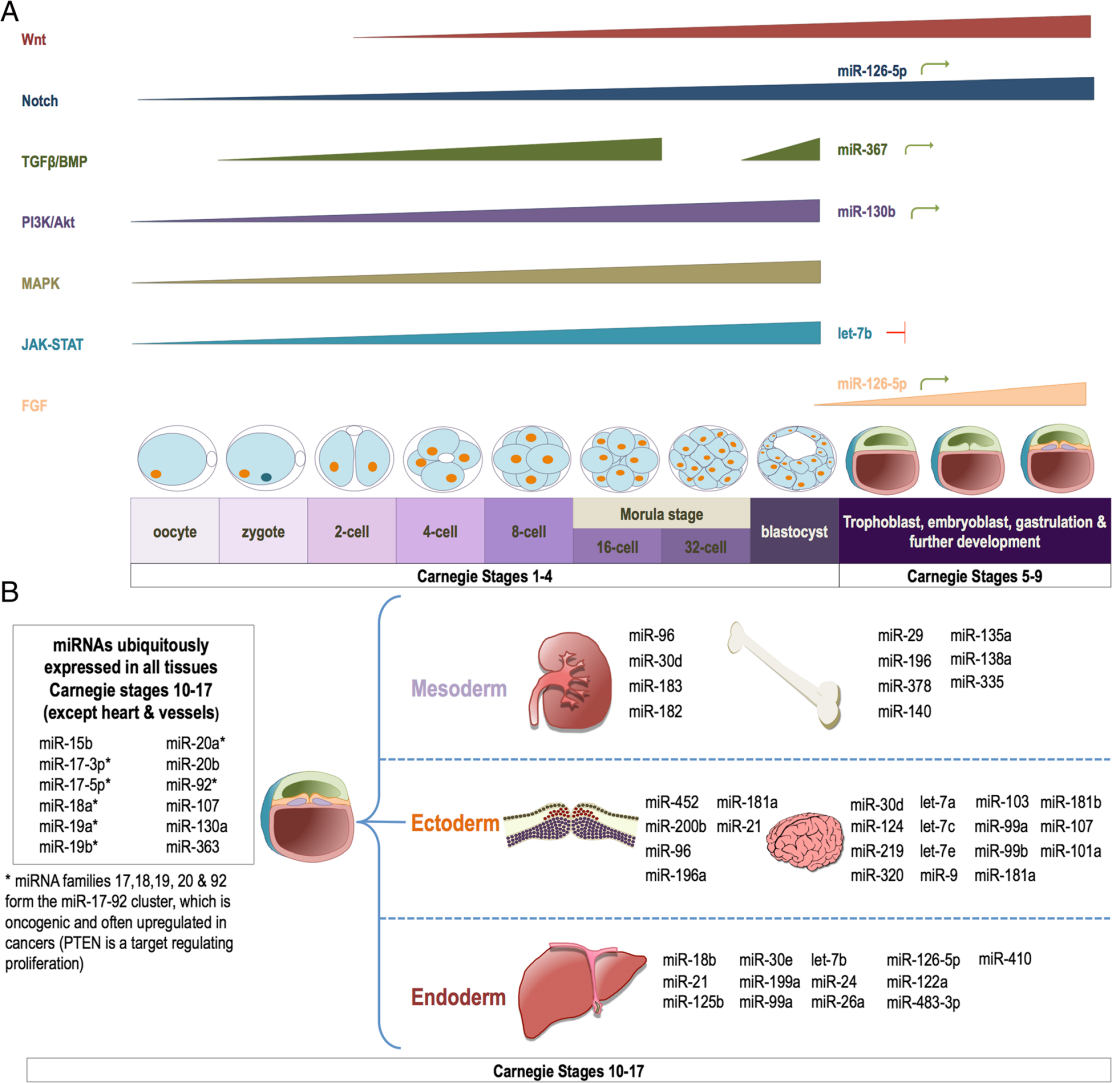
Proposed schematic of early developmental processes linking key signalling pathways and miRNA expression. **a** The developmental signalling pathway expression in early stages of development (Carnegie stages 1–9), and the miRNAs demonstrated to regulate these pathways during these stages. **b** Carnegie stages 10–17, representing further development and miRNAs ubiquitously expressed in all tissues (except heart and vessels). The three germ layers are represented and their associated body systems and organs are discussed in a cancer context in this review (kidney; WT, bone; OS, Neural crest; NB, brain; embryonal brain tumours, liver; HB). Additionally miRNAs associated with each organ/body system are presented

In the earliest embryonic stages, a single cell zygote transforms to a morula and then to a blastocyst. During this period the zygote initiates the first cell division and the first lineage cell undergoes differentiation into the inner cell mass and the trophectoderm. The processes involved in these events are complex and regulated by multiple signalling pathways with related biological functions such as cell division and growth, differentiation, migration, apoptosis, transformation and polarity. Not surprisingly these functions are tightly interconnected and disruptions in these networks may lead to abnormal development, disease or even fatality. Several signalling transduction pathways have been demonstrated to be involved in the early process of mammalian embryonic development, including; mitogen-activated protein kinase (MAPK); phosphatidylinositol 3-kinase (PI3K)/Akt; Wingless (Wnt)/β-catenin; Notch; bone morphogenetic protein (BMP)/transforming growth factor (TGF- β); Hedgehog and Janus-activated kinase (JAK)/signal transducer and activator of transcription (STAT). While these pathways contribute to central events in development, their stage-specific expression is key to orchestrating the correct processes with the correct timing; this is where miRNAs may have an important role. The miRNA expression in preimplantation development is a difficult area to research, particularly in humans and therefore much of this information is still relatively unexplored. However miRNA targeting of developmental pathways does occur early in development and the identified action of specific miRNAs targeting critical signalling pathways provides evidence of the importance of these molecules in regulating early development and assisting in more specified development in separate lineages, and body systems later on (Fig. 1) [47].

There are a large number of miRNAs that have been confirmed to regulate components in essential developmental pathways. Crosstalk between these signalling pathways and miRNAs emphasises that miRNAs contribute largely during embryogenesis and early development. An example is the Wnt signalling pathway, which is a major contributor in controlling biological processes and is extensively researched. There is supportive evidence of miRNAs regulating WNT signalling components and being associated with various cancers in the literature. The signalling pathways critical in early development are interwoven with the function of numerous miRNAs and the affect of aberrant miRNA expression could have widespread consequences in the development of a specific organ or body system. Additionally, mice lacking the essential miRNA-processing enzyme, Dicer, are embryonically lethal before embryonic day (E) 7.5, suggesting the roles of the components of the machinery which produce functional miRNAs, and the miRNAs themselves, are essential in embryogenesis [48].

### miRNA function and links to childhood cancer development and progression

**Fig. 2:**
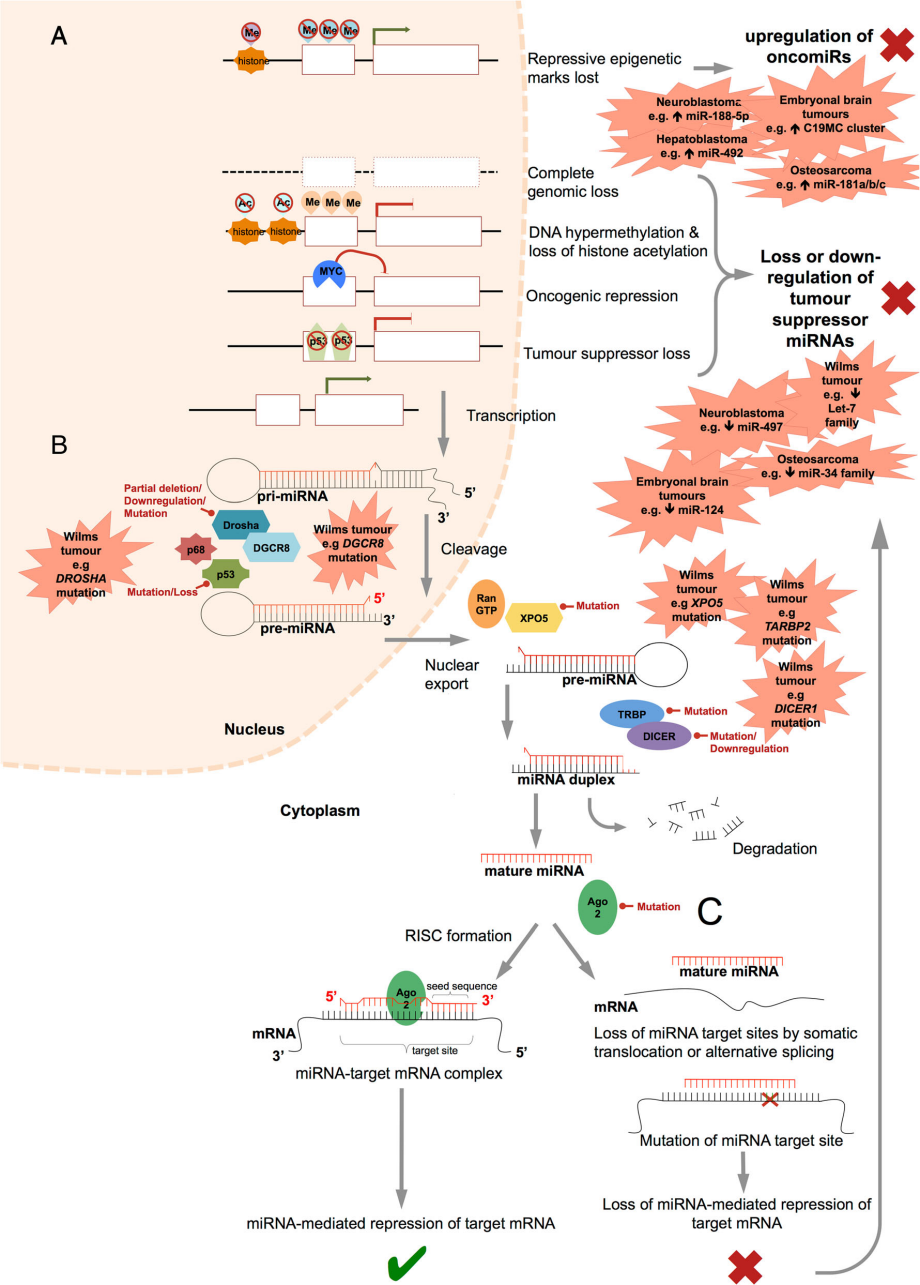
The mechanisms by which alteration of normal miRNA profiles can occur, leading to aberrant miRNA profiles in childhood solid tumours. Examples of aberrant expression and disrupted miRNA biogenesis are illustrated and are supported by validation in the literature. **a** Disruptions to miRNA genes, resulting in upregulation of oncogenic miRNAs or downregulation, or complete loss of expression (tumour suppressor miRNAs). **b** Components of the miRNA biogenesis pathway that can be altered, affecting production of mature functioning miRNAs. **c** Events which alter the target mRNA and affect the ability of the mature miRNA to bind

There are a number of mechanisms that may affect the miRNA profile in a specific tissue or group of cells. These events can alter expression in both oncomiRs and tumour suppressor miRNAs, or on a more global scale, mutations in components of the miRNA biogenesis pathway may affect larger groups of miRNAs, and their ability to mediate repression of target mRNA (Fig. 2.) The let-7 family were identified early as a group of influential miRNAs. They are well researched and have been shown to regulate expression of the *RAS* oncogenes*.* Mutations in *RAS* oncogenes are present in around 25–30% of all human tumours. *In vitro* experiments on a pulmonary adenoma cell lineage indicate that let-7 is able to inhibit cell proliferation through *RAS*, which suggests it may play a role as a tumour suppressor [49]. From this investigation, it was determined that let-7 miRNAs regulate the expression of the RAS protein and are also able to subsequently affect cell proliferation rates through the downstream MAPK signalling cascade.

Although the links between miRNAs and the initiation and progression of cancer are supported, the area which remains difficult to interpret and requires further exploration is the exact mechanism by which miRNAs function to cause downstream effects, or conversely, how miRNA genes are influenced by other events which may alter the normal miRNA profile. Often miRNA expression is repressed in cancer compared to normal tissues; contrarily, there are also some miRNAs overexpressed in cancer [24].

In addition to the deletion and mutation of miRNA genes, aberrant methylation may cause epigenetic inactivation with subsequent transcriptional suppression. Aberrant hypermethylation in mir-9-1, mir-124a, mir-148, mir-152 and mir-663 was observed in up to 86% of cases in a cohort of 71 primary breast cancer tissues [50]. Additionally, global or local aberrant methylation of miRNA genes may also alter the miRNA profile of an individual, subsequently providing a vulnerable situation in which normal genetic regulation is lost and the development of cancer could arise. In relation to this, epigenetic changes of miRNA promoters, such as alteration of their methylation status or their histone patterning can also induce changes in miRNA expression [51]. A number of studies have investigated the role of methylation and how it may fit in with disease progression and development. What is important to note here, and can be applied to other types of cancer, are the mechanisms by which epigenetic regulation can occur. Some examples of this include the downregulation of Let-7 family, which has been shown to be downregulated through DNA and histone methylation in gastric cancer, or miR-34b and miR-34c. Or miRNAs can be downregulated through hypermethylation of the neighbouring CpG islands. These miRNAs are also implicated in childhood cancers suggesting a common role in oncogenesis [52] (Table 1). Accumulating evidence suggests that aberrant methylation of certain miRNA genes may disrupt a number of key signalling pathways. This could have major effects in the developmental process, which relies heavily on the maintenance of regulated signalling to ensure controlled growth, and without which tumourigenesis may arise (Fig. 2).

**Table 1:** Summary of miRNAs that are consistently downregulated in different childhood solid tumours

miRNA/miRNA cluster		Childhood cancer type*	Comments	Ref
miR-16		OS	Differentially expressed compared to healthy bone tissue	[103]
miR-29b		OS	Differentially expressed compared to healthy bone tissue	[103]
miR-142-5p		OS	Differentially expressed compared to healthy bone tissue	[103]
miR-34 family	miR-34a, miR-34b, miR-34c	NB, OS	Targets *MYCN,* identified as tumour suppressors, often found in 1p deletion in MNA NB tumours, targeted by p53	[79], [82], [84], [97–101]
miR-451		OS	Higher expression in pre-treatment samples, corresponded with positive response to chemotherapy	[103]
miR-15b		OS	Higher expression in pre-treatment samples, corresponded with positive response to chemotherapy	[103]
miR-125b-1-3p		NB	Lower expression may be associated with chemoresistence	[86]
miR-199a-3p		OS	Differentially expressed in OS cell lines, regulation of proliferation in osteoblasts, acts as tumour suppressor	[96]
miR-127-3p		OS	Differentially expressed in OS cell lines, regulation of proliferation in osteoblasts, acts as tumour suppressor	[96]
miR-370		OS	Differentially expressed in OS cell lines, regulation of proliferation in osteoblasts, acts as tumour suppressor	[96]
Let-7 family	Let-7a, Let-7b, Let-7c, Let-7d, Let-7e, Let-7f, Let-7 g, Let-7i, miR-98	WT	Impaired expression observed due to *DICER1* and *DROSHA* mutations, regulators of *MYCN* and *LIN28*	[28] [113]
miR-195		HB	Differentially expressed in fetal subtype compared to surrounding normal liver tissue	[121]
miR-210		HB	Differentially expressed in fetal subtype compared to surrounding normal liver tissue	[121]
miR-214		HB	Differentially expressed in fetal subtype compared to surrounding normal liver tissue	[121]
miR-124		PA	Differentially expressed in PA tumours compared to normal brain tissue, targets putative oncogenes, enriched in brain tissue	[66], [67]
miR-218		MB	Low expression observed, targets include pathways involved in cell cycle, metabolism and motility, *CDK6* is target of interest (upregulates cell cycle progression and blocks differentiation)	[71], [72]
miR-497		NB	Targets key cell cycle regulator *WEE1.* Low levels seen in high risk MNA tumours which also display high levels of *WEE1.* It is a candidate tumour suppressor and may be useful as a therapeutic target.	[85]

*NB neuroblastoma, MB medulloblastoma, *OS* osteosarcoma, *WT* Wilms Tumour, *HB* hepatoblastoma, *PA* pediatric pilocystic astrocytoma

Cancer-related transcriptional control of miRNAs has also been demonstrated by the observation of transcriptional activation of the *miR-17/92* cluster induced by the *MYC* oncogene, the upregulation of which modulates the anti-apoptotic and proliferative actions of E2F1, mediating the *MYC* proliferative effect [13]. Altered miRNA expression clearly contributes to many cancers through a variety of different mechanisms, and will continue to provide insight into cancer onset and progression.

### A brief account of childhood solid tumours

In childhood cancers, the pathogenesis of tumours is often tightly linked with the processes of development such as organogenesis, tissue growth and maturation [53–58]. This area of research is growing; new resources collating and examining the links between developmental biology and oncology are emerging to assist in bringing together the two research communities to help apply discoveries in the search for targeted therapies [59]. The processes regulating fetal and postnatal growth and development are controlled and coordinated by multiple developmental pathways, such as Hox, WNT, Notch and Hedgehog [47]. Disruption of these strictly regulated pathways through germline or somatic mutation, or epigenetic or transcriptional dysregulation can transform specific tissues, giving rise to the early onset of cancer during childhood [30].

The most common types of childhood cancers are leukaemia, brain and central nervous systems tumours and lymphomas. In addition to these cancers, are the embryonal solid tumours of the kidney (Wilms tumour), neural crest (neuroblastoma), muscle (rhabdomyosarcoma), eye (retinoblastoma), and the liver (hepatoblastoma). Embryonal solid tumours of childhood are more commonly sporadic than heritable. In some cases they are associated with various syndromes such as Beckwith-Wiedemann syndrome (BWS) and in patients with familial adenomatous polyposis (FAP) [60]. The particular histological features of the malignant tumours, and the appearance of corrupted tissue development, are what characterise this heterogeneous group of tumours.

### The role of miRNAs in childhood cancer

It is essential to continue research to build a better understanding of the role miRNAs in the development of childhood cancers. It is clear that many of these cancers are tightly associated with irregular events occurring during development, and what we can learn from the inclusion and exploration of these vital regulatory elements may be the key to a deeper understanding of the complex nature of how childhood cancers arise. Another complex issue involving childhood cancers, where miRNAs may provide valuable insight, is the development of treatment resistance. It is important to identify aberrant miRNA expression associated with pathogenesis and also drug resistance, especially chemoresistance, as this may be critical in providing lasting and effective treatments for children [61].

The following sections review several types of childhood solid tumours, describing common features and key developmental pathways that have been identified to contribute to embryogenesis and disease. The following childhood cancers were selected based on their association with a variety of affected organs or body systems, (e.g. kidneys, liver and brain as well as the sympathetic nervous system and skeletal system), and because recent literature has uncovered unique links to miRNAs and the development of these solid tumours. In addition, the childhood cancers discussed are those where the etiology remains unclear and the involvement of miRNAs could help to explain some of the unique qualities of the disease and may allow for a better understanding of new opportunities for targeted therapies. Young children are most at risk for developing debilitating late effects from the regimented chemotherapy courses. Through knowledge gained from these studies, we may be able to make better predictions about prognosis.

#### Embryonal tumours of the brain

A number of studies in recent times have indicated the frequent involvement of miRNAs in the progression and development of childhood brain tumours, and that this tumour has unique characteristics among rare tumour types. Brain tumours are among the most common cancers in children. The different tumour types and classifcations are generally based on cell structure, composition, growth rate of the tumour and a range of further characteristics. Central nervous system primitive neuro-ectodermal brain tumours (CNS-PNETs) are a heterogeneous group of CNS neoplasms. These tumours are composed of poorly differentiated neuroepithelial cells and, some variants are associated with aggressive clinical behaviour and poor outcome.

Chromosome 19q13.41 contains the largest human miRNA gene cluster (chromosome 19 miRNA cluster, C19MC); the miRNAs comprising this cluster have been identified as oncogenic [62]. C19MC may drive oncogenic processes in part by facilitating maintenance and transformation of a very early neural compartment [63, 64]. A particularly aggressive group of CNS-PNET tumours demonstrated C19MC amplification with high *LIN28* expression. In addition, recent identification of Tweety family member 1 (*TTYH1*):C19MC gene fusions in embryonal tumours with multi-layered rosettes (ETMRs) have been identified and observed to be associated with very high expression of specific miRNAs. ETMRs are a rare and deadly form of paediatric brain tumours, and are characterized by high levels of amplification of C19MC. ETMRs, cell lines and xenografts all demonstrated specific DNA methylation signatures distinct from other tumours and normal tissues, very high overexpression of a previously uncharacterized isoform of *DNMT3B,* which originates from an alternative promoter, and which is interestingly only active in the first weeks of neural tube development. This research uncovers a potential oncogenic re-activation of an early developmental signalling program in ETMR via an epigenetic alteration mediated by the brain specific *DNMT3B* isoform, which has a known embryonic origin [65].

Pediatric pilocystic astrocytoma (PA) is a Word Health Organisation grade I glioma. It is a brain tumour, which often arises in the cerebellum or close to the brainstem in the hypothalamic region or the optic chiasm. However it is possible for it to occur in any location where astrocytes are present (cerebral hemispheres, and the spinal cord). While often these tumours are slow growing and benign, the neoplasms can be cystic and grow very large, causing multiple problems due to the increased intracranial pressure. miRNA profiling has been performed in a group of 43 PA tumours and 5 non-neoplastic brain controls. Differentially expressed miRNAs were identified between PA and normal brain tissue; 13 miRNAs were underexpressed in PA and 20 were over expressed compared to normal brain tissue (Table 1). An example of the importance of a few of these differentially expressed miRNAs is miR-124, which was underexpressed in PA tumours, confirming previous findings. miR-124 targets putative oncogenes, is enriched in brain tissue, and is also found to be downregulated in glioblastomas. miR-124 also has been shown to negatively affect glioblastoma proliferation and migration in vitro [66, 67]. Additionally, upregulation of miR-21 was shown in these tumours and elevation of this miRNA has also been seen in other tumour types, compared with normal tissues (Table 2). An important target of miR-21 is PTEN, a critical suppressor of the PI3K/Akt/mTOR pathway. Previously PTEN loss had been frequently identified in high-grade gliomas, and decreased levels had been shown in PAs with particularly aggressive histological features. An overall observation of miRNAs in PA is that there are sets of miRNAs underexpressed compared to controls, and alongside this there is increased expression and protein levels of putative oncogenes which may be vital in understanding the biology of PA [68, 69].

**Table 2:** Summary of miRNAs that are consistently upregulated in different childhood solid tumours

miRNA/miRNA cluster		Childhood cancer type*	Comments	Ref
miR-27a		OS	Higher expression in pre-treatment samples characterized metastatic disease	[103]
miR-188-5p		NB	Higher expression may be associated with chemoresistance	[86]
miR-501-5p		NB	Higher expression may be associated with chemoresistance	[86]
miR-135b		OS	Differentially expressed in OS cell line compared to normal osteoblast cell line, association with osteoblast differentiation	[91–95]
miR-150		OS	Differentially expressed in OS cell line compared to normal osteoblast cell line	[91]
miR-542-5p		OS	Differentially expressed in OS cell line compared to normal osteoblast cell line	[91]
miR-652		OS	Differentially expressed in OS cell line compared to normal osteoblast cell line	[91]
miR-181a		OS	Differentially expressed compared to healthy bone tissue	[103]
miR-181b		OS	Differentially expressed compared to healthy bone tissue	[103]
miR-181c		OS	Differentially expressed compared to healthy bone tissue	[103]
miR-492		HB	Elevated levels co-expressed with KRT19, a potential biomarker for metastatic HB and poor prognosis	[120]
miR-222		HB	Low relative expression associated with increased overall survival of HB	[121]
miR-224		HB	Low relative expression associated with increased overall survival of HB	[121]
miR-221		HB	Differentially expressed in fetal subtype compared to surrounding normal liver tissue	[121]
miR-483-3p		HB	High overexpression in tumour specific serum able to diagnose liver mass	[123]
miR-205-5p		HB	High overexpression in tumour specific serum able to diagnose liver mass	[123]
C19MC	miR-512-5p, miR-512-3p, miR-1323, miR-498, miR-520e, miR-515-5p, miR-515-3p, miR-519e-5p, miR-miR-519e-3p, miR-520f, miR-1283, miR-520a-5p, miR-520a-3p, miR-526b-5p, miR-526b-3p, miR-519b-5p, miR-519b-3p, miR-525-5p, miR-525-3p, miR-523-5p, miR-523-3p, miR-518f-5p, miR-518f-3p, miR-520b	CNS-PNET ETMR	C19MC amplification seen in both CNS-PNET and ETMRs, miRNA members are considered oncogenic and may maintain transformation of a very early neural compartment. In CNS-PNET C19MC amplification is observed with high LIN28 expression. In ETMRs TTYH1:C19MC gene fusions are observed with very high expression of miRNA members from this cluster.	[63], [64], [65]
miR-106b		ET	Associated with the progression of ET from grade II to grade III, potential prognostic marker	[70]

*NB neuroblastoma, *OS*-osteosarcoma, *HB* hepatoblastoma, *CNS* *PNET*-central nervous system primitive neuro-ectodermal brain tumour, *EMTR* embryonal tumours with multi-layered rosettes, and *ET* ependymal tumour

Ependymal tumours (ET) are the third most common group of brain tumours in children. These tumours comprise four entities; the most common are grade II and grade III, most of which are located in the posterior fossa, with infiltration into vital brain structures. Due to the difficult location of these tumours surgical resection is highly limited. Additionally there are currently no effective prognostic features aside from how accessible they are to surgical intervention. Childhood ependymomas usually don’t have genomic imbalances, further making it difficult to establish molecular prognostic features. Recently, however, three miRNAs were demonstrated to differentiate between grade II and III ependymomas. These include miR-17–5p, miR-19a–3p and miR-106b–5p, and the expression of these miRNAs was also significantly correlated with EZH2 expression (a suggested marker for PFA ependymomas). Survival analysis indicated overall and event free survivals were reduced with higher expression levels of miR-17–5p [70].

Medulloblastomas (MBs) are a common brain tumour in children, thought to originate in cerebellar granule neuron progenitors, which have failed to undergo normal cell cycle exit and differentiation. In MB miR-218 expression is downregulated, and low expression of this miRNA has been identified in other cancer types. In addition, reduced expression has been observed in glioma cells [71] (Table 1). Previous research has identified multiple targets of miR-218 that control pathways involved with the cell cycle, cell metabolism and motility. Of those identified, *CDK6* is of interest as it has previously been identified as an adverse prognostic marker in MB because of its role in upregulating cell cycle progression and blocking differentiation [72]. The *miR-17-92* cluster is also implicated in MB. Mouse MB models have demonstrated overexpression of several members of the *miR-17-92* cluster, three of which, (miR-19a, miR-20, miR-92), were also overexpressed in human MBs with a constitutively activated Sonic Hedgehog signaling pathway, and not in other forms of the disease (Table 2). To explore whether the *miR-17-92* cluster could be promoting MB formation, the expression of these miRNAs was enforced in granule neuron progenitors isolated from cerebella of postnatal day six mouse models (*lnk4c−/−; ptch1+/−*). These mice formed MBs in orthotopic transplants with complete penetrance, but in similarly engineered cells from *lnk4−/−; p53−/−* mice there was no MB formation. These findings point toward a possible functional connection between the *miR-17-92* cluster, the Sonic Hedgehog signaling pathway, and the development of MBs in both mice and humans [73].

#### Neuroblastoma

Neuroblastoma (NB) is an extracranial solid childhood tumour, and is the most common cancer in infants. The cancer is a neuroendocrine tumour, stemming from neural crest elements of the sympathetic nervous system; it is an ectodermally derived tumour, which reflects its developmentally derived origin [59]. While it frequently originates in one of the adrenal glands, it can also develop in nerve tissues in the neck, chest, abdomen or pelvis [74]. It is a unique and interesting cancer, as it is one of the few malignancies that can demonstrate spontaneous regression from an undifferentiated state to a benign cellular appearance; additionally, there is a large amount of heterogeneity observed in these tumours [75].

Recent work in this area suggests the dysregulation of miRNAs may play an important role in the pathogenesis of NB. For instance, the *miR-17-5p-92* cluster of miRNAs, (miR-17-5p, miR-18a, miR-19a, miR-20a and miR-92), is found to be expressed at higher levels in NB cells lines exhibiting overexpression of *MYCN* [76]*.* Tumours with amplification of the MYCN transcription factor and/or loss of the distal chromosome 1p and gain of 17q are a major genetic subtype of metastatic NB that has a particularly poor prognosis (known as MNA). It has also been demonstrated that MYCN proteins c-Myc/N-Myc can bind directly to the promoter of the miR-17-5p-92 cluster, initiating transcription and resulting in the up-regulation of this group of miRNAs from a single transcription unit [77, 78]. A poor prognosis and therapy resistance is associated with *MYCN* amplified NBs. However, previous research has shown that therapy resistant NB can be abolished in vivo using antagomir-17-5p [76].

Components within the miRNA biogenesis pathway have also been implicated in NB, which globally affects miRNA expression. A group of 66 tumours were assessed for a panel of 162 miRNAs using quantitative PCR and low expression of *DICER* and *DROSHA* were identified in high-risk tumours. The low expression subsequently accounted for the overall global reduction of miRNA expression observed in the advanced disease state and correlated with a poor outcome for these patients [26]. A global reduction of miRNA expression can be detrimental, and conversely the presence of some miRNAs has been attributed to more positive outcomes, such as the tumour suppressor miR-34a, which is also a critical component of the p53 network [79, 80]. This miRNA has anti-proliferative effects and has been found to target *MYCN.* *miR-34a* is located on chromosome 1 and it is not surprising that lower levels are expressed in tumours with/or due to a 1p deletion [81]. This type of deletion is found most often in the MNA type of tumours; a functionally active miR-34a would help to negate the ill effects of the amplification of *MYCN.* miR-34a is also involved in the regulation of other genes associated with cell proliferation and apoptosis such as *E2F3, BCL2, CCND1,* and *CDK6* [82, 83]. Since miR-34a plays many roles in controlling and regulating growth, it is obvious disruption of the function of this miRNA would be deleterious and could lead to unrestricted growth leading to cancer development, and indeed miR-34a has been described previously as a tumour suppressor [79, 84]. Another miRNA that has been reported as a potential tumour suppressor is miR-497; this is due to its action in targeting the key cell cycle regulator *WEE1*. Expression of miR-497 in MNA tumours is significantly lower than other tumour types, and high *WEE1* levels (low miR-497) are significantly associated with poor overall survival and event free survival in NB (Table 1). Additionally, overexpression of miR-497 reduces cell viability and increases apoptosis in MNA cells, and in particular was found to be compatible with an enhanced response to cisplatin. The discovery of the complexity involved in miRNA control has begun to uncover new and promising therapeutic targets for high-risk NB patients [85].

Another common issue seen in childhood solid tumours is chemotherapy resistance; one study investigated drug resistance and expression of miRNAs, and identified several miRNAs of interest in NB chemoresistance models. Their results confirmed an up-regulation of the expression of miR-188-5p in all three of the chemoresistance models examined; miR-501-5p was found up-regulated, and miR-125b-1 was down-regulated in two of the models [86]. These types of investigations are valuable in identifying good miRNA candidates for in-depth exploration to look for novel drug development and perhaps RNAi based therapies for those individuals who are not responding to chemotherapy and may have more aggressive tumour types [87, 88].

#### Osteosarcoma

Osteosarcoma (OS) is an aggressive malignant neoplasm of the bone that originates in primitive transformed mesenchymal cells [89]. This cancer begins in osteoblasts responsible for forming new bone and is common in adolescents. Bone formation is a dynamic process; orchestrating osteoblast differentiation, which requires precise mechanisms of control, and is particularly critical for both development and growth. OSs tend to occur at the sites of bone growth, and the proliferation occurring at these locations may make the osteoblastic cells more susceptible to acquiring mutations, or other events, which may induce the transformation of cells [90]. Therefore, often the ends of long bones (arms and legs) are affected, although formation in the knee is also a common event.

The molecular etiology of OS remains elusive; miRNAs have been investigated in association with many diseases due to their properties that may allow for widespread effects on development and disease progression. In prior investigations four miRNAs were found to be differentially expressed in OS cell lines compared to normal human osteoblast cell lines [91]. The miRNAs that were overexpressed in the OS cell lines included, miR-135b, which has previously been implicated in cancer and is associated with osteoblast differentiation, miR-150, miR-542-5p and miR-652 [92–95]. Several miRNAs have been identified that may act as tumour suppressors in OS and are additionally found to be down-regulated in OS cell lines. These miRNAs, (miR-199a-3p, miR-127-3p and miR-370), assist with the suppression of oncogenic and anti-apoptotic proteins and also play a role in regulating proliferation of the osteoblasts [96]. As with NB, the miR-34 family is again highlighted as an important regulator of growth signals and acts as a tumour suppressor miRNA. These miRNAs are targeted by p53 and play a very important part of the tumour suppressor pathway [82, 97–102].

In a recent investigation, a miRNA signature reflecting the pathogenesis of OS was described using surgically procured samples from human patients [103]. These samples highly expressed miR-181a, miR-181b and miR-181c and also showed reduced expression of miR-16, miR-29b and miR-142-5p. However, it is noteworthy this study also identified valuable pre-treatment biomarkers of metastasis and indicators of responsiveness to therapy. The biomarkers and indicators of metastatic development risk included a higher expression of miR-27a and miR-181c-3p in the pre-treatment samples. This characterised patients who went on to develop metastastic lesions. Furthermore, higher expression of miR-451 and miR-15b in pre-treatment samples correlated with positive responses to chemotherapy [103].

#### Wilms tumour

Wilms tumour (WT), or nephroblastoma is a solid tumour occurring in the kidneys of children. Embryonal renal neoplasms are thought to develop in nephrogenic rests (NRs) with morphological and molecular analogies to kidney development. Most commonly WTs are unilateral with a single tumour affecting the kidney. However multiple tumours can arise in one of the kidneys, or the cancer may occur bilaterally in both kidneys [104, 105].

In WT relatively few driver genes have been identified [106]. Recently both Sanger sequencing and whole exome sequencing have provided the means to identify mutations in critical elements of the miRNA processing machinery in WT patients. Several recent studies have uncovered miRNA biogenesis disruptions in WT, which provides an excellent starting point for research in this area [27, 28, 107]. Rakheja and colleagues investigated the presence of these potential mutations in 44 WT samples using whole-exome sequencing [28]. In addition to identifying novel mutations in *MYCN, SMARCA4* and *ARID1A*, missense mutations were discovered in the miRNA processing enzymes *DROSHA* and *DICER1.* Further examination of tumour miRNA expression using in vitro processing assays and genomic editing of cell lines demonstrated that these mutations in *DROSHA* and *DICER1* greatly influence miRNA processing and likely cause down regulation in mature miRNAs, which generate an altered miRNA expression profile in these WT patients. Further, there were distinct mechanisms also identified; *DICER1* RNase IIIB mutations preferentially affected the processing of miRNAs derived from the 5’ arm of pre-miRNA hairpins. Conversely *DROSHA* RNase IIIB mutations were found to cause global inhibition of miRNA biogenesis through a dominant-negative mechanism. Mutations in these enzymes also impaired the expression of tumour suppressor miRNAs such as the let-7 family; this is an important regulator of genes such as *MYCN, LIN28* as well as other previously identified WT oncogenes [28]. Downregulation of certain miRNAs in WT were also observed to have a major affect on critical pathways that are involved in kidney development, such as, TGF-β. For example Activin A receptor type 2B (ACVR2B), an important component of the TGF-β pathway, is often highly expressed in renal neoplasms; several miRNAs have been noted as downregulated and have been confirmed to directly target this gene (Table 1). This investigation was the first to implicate the TGF-β pathway in the pathogenesis of WT [108].

A number of up regulated miRNAs were also identified in WT; up-regulation of miR-17-5p, miR-18a, miR-19b, miR-92 and miR-20a was reported in WT compared to other kidney tumours or normal kidney tissues [109]. This is of interest, because the cluster (*miR-17-5p-92*) was also identified as being up-regulated in the subtype of NB known as MNA [76] (Table 3). As mentioned, these miRNAs are thought to contribute to tumour progression and metastasis in the later stages of the cancer [103, 110]. Other miRNAs found to be upregulated in WT have been investigated for being able to distinguish the different subtypes of WT and for giving an indication on severity of disease, or for influencing important factors such as endothelial to mesenchymal transitions (EMT) and chemosensitivity [111]. Another mechanism recently recognised in the development of WT is the aberrant regulation of miRNA Let-7 through overexpression of *LIN28* [112]. Let-7 is a direct target of *LIN28*, and it is a potent regulator of stem cell self-renewal and differentiation. A murine model has shown that directed overexpression of both *LIN28A and LIN28B* in the renal lineage results in the formation of WTs [113]. Further investigation of *LIN28* expression in mutant mice revealed that terminal differentiation only occurred in nephron progenitor cells after *LIN28* had been completely withdrawn. It is plausible that the overexpression of *LIN28* may cause an imbalance in proliferation and differentiation in these specific cells, rather than a complete blockage of differentiation, resulting in the formation of these types of tumours [113, 114].

**Table 3:** Summary of miRNAs that are either up or down regulated or contains conflicting evidence of expression in childhood solid tumours

miRNA/miRNA cluster		Childhood cancer type^*^	Comments	Ref
miR-17-92 cluster	miR-17-5p, miR-18a, miR-19a, miR-19b, miR-20a, miR-92	NB, WT, MB, ET (upregulated)	c-Myc/n-Myc bind to increase expression in *MYCN* amplified NB	[70, 76–78, 109, 121]
		HB (downregulated)	Upregulation in WT compared to other kidney tumours or normal tissue	
			Lower expression of miR-17-5p in fetal subtype of HB compared to surrounding non-tumourous liver tissue	
			Overexpressed in mouse MB models and human MBs (some with constitutively activated Sonic Hedgehog signalling)	
			High levels of expression of miR-17-5p and miR-19a-3p associate with grade III (advancement) in ET	
miR-21		HB (Downregulated)	High relative expression associated with increased overall survival of HB	[121]
		PA (upregulated)	Upregulation seen in PA with more aggressive histological features, an important target is PTEN	
miR-122-5p		HB (upregulated, dowregulated)	miR-21 was detected in higher levels in HB patients in both the plasma and exosomes compared to control patients.	[25, 122–124]
			Low expression of miR-122-5p is seen in the embryonal subtype of HB. Serum miRNA profiles of HB patients indicated high expression of miR-122-5p and could be used in a panel to perform a non-invasive differential diagnosis of liver mass.	

^*^ *NB*neuroblastoma, *MB*medulloblastoma, *WT* Wilms’ Tumour, *HB* hepatoblastoma, *PA* pediatric pilocystic astrocytoma, and *ET* ependymal tumour

#### Hepatoblastoma

Hepatoblastoma (HB) is a relatively rare disease, although it is the most common childhood liver cancer. It is often diagnosed as an asymptomatic abdominal mass [115]. The most commonly affected age group are infants between six months and three years of age. The majority of HB cases are sporadic. However, HB may occur in conjunction with a developmental syndrome such as Beckwith-Wiedemann Syndrome (BWS) and Familial Adenomatous Polyposis (FAP) [116, 117].

Since HB is a rare cancer, research is challenging and often relies on formalin fixed paraffin embedded (FFPE) samples, which are known to provide obstacles in the extraction of total RNA due to degradation. However, recent research has demonstrated miRNA expression is relatively stable and well preserved in these valuable archival samples which is an important factor for future research in this field [118, 119]. Previous studies provided evidence for a few prognostic miRNAs associated with HB, such as miR-492, which is a potential biomarker in metastatic HB. Overexpression of *pleiomorphic adenoma gene 1* (*PLAG1*) has been characterised in HB, and has been demonstrated through RNA interference analysis combined with miRNA arrays to strongly influence miR-492. Additionally, it was revealed that miR-492 could originate from the coding sequence of the HB marker gene keratin 19 (*KRT19*). Significantly elevated levels of co-expressed *KRT19* and miR-492 were identified in metastatic HB tumour samples [120]. This is of interest as metastatic HB is often associated with poor prognosis. Indeed, miR-492 and its associated targets may provide biomarkers or inform on targeted therapies [120].

Other potential prognostic miRNAs have been identified in a recent study, which showed that histological subtypes of a group of 20 tumour samples did not correlate with survival. However this investigation did identify that the levels of several miRNAs were independently prognostic for HB with significantly increased overall survival. These miRNAs included the high relative expression of miR-21 and low relative expression of miR-222 and miR-224 [121] (Tables 2 and 3). In a recent study miR-21 was examined for its potential role as a diagnostic/prognostic indicator in peripheral blood where it has been reported to be relatively stable within protective exosomes or nanovesicles. Exosomes, which can be released in large amounts from tumour cells due to a hypoxic environment and other internal changes, are of interest because a blood sample is a less invasive method for diagnosis and prognosis for the patient. Blood samples were prepared retrospectively in 32 Chinese hepatoblastoma patients and healthy controls; the blood samples were separated to isolate RNA directly from the exosomes present in the sample (which precipitate at the bottom), while also preparing exosome-depleted samples (using the supernatant of the same sample). The concentration of miR-21 in the exosomes prepared from blood of HB patients was significantly higher than in exosome-depleted supernatants and whole plasma. The expression of miR-21 detected in HB children was significantly higher in both the plasma and exosomes when compared to controls. Additionally, exosomal miR-21 is not only an independent predictor of event-free survival for patients, it also was more accurate in diagnosing HB than alpha-fetoprotein levels (AFP), the traditional means of HB diagnosis [122]. There were also miRNAs identified with unique expression levels in both fetal and embryonal subtypes in comparison with surrounding non-tumorous liver tissues. In the fetal subtype of samples, there was a lower expression compared to surrounding normal liver tissue in miRNAs miR-17-5p, miR-195, miR-210 and miR-214, while higher expression was observed in miR-221 (Tables 1, 2 and 3). In the embryonal subtype of HB a lower expression of miR-122-5p was demonstrated. Loss of miR-122-5p is also frequent in hepatocellular carcinoma (HCC) and has been correlated with migration, invasion, and in vivo tumorigenesis. miR-122-5p is also considered a differentiation marker for hepatocytes. Lower miR-122-5p expression levels indicated in the embryonal subtype is in agreement with the lower degree of differentiation found in early embryonal development. In a recent comprehensive investigation of 33 different types of childhood solid tumours and 20 control cases, serum miRNA profiles were examined. Four HB samples were included in this group of tumours and tumour specific serum miRNA profiles were determined for each specific tumour type. Using the panel of miRNAs identified for HB, (high overexpression of miR-483-3p, miR-205-5p, and miR-122-5p), a non-invasive differential diagnosis of a liver mass could be performed when compared to tumour mixed samples of neuroblastomas (*MYCN*-amplified and others) [25]. It is of interest that contradicting results from two studies identified miR-122-5p as being of interest, except at opposite ends of the spectrum, (one observing lower relative expression and the other stating the miRNA to be in the top ten of the highest relative overexpression) (Table 3). It is important to acknowledge that in serum miRNA profiling investigations only four HB samples were investigated, and in addition the authors pointed out that miR-122-5p was found in increased levels in non-HB samples in this study, such as in pancreatic pleiomorphic rhadomyosarcoma (RMS) presenting with obstructive jaundice [25]. Serum levels of miR-122-5p were increased, while the expression of miR-122-5p was reduced in liver tissue, which could be due to passive miRNA drainage from tumour cells, as has been observed in other tumours [123]. Expression of miR-122-5p has been identified as a non-specific marker of liver damage, and is increased in the serum of jaundiced patients [124]. These studies highlight the importance of identifying miRNA profiles (in tumour tissue and in serum), and shows that there may be specific miRNAs with unique roles in various types of childhood solid tumours.

## Conclusions

The role of miRNAs in the regulation of critical signalling pathways such as Wnt/β-catenin, Notch, and TGFβ/BMP, has emerged as an important area in the exploration of childhood cancer development. Childhood solid tumours often display heterogeneous embryonic features, pointing to an early origin of tumourigenesis. While research into miRNA expression at very early stages of embryonic development (preimplantation) is largely unexplored at this stage, there is evidence that miRNAs regulate key components of several developmental pathways following these stages, and that expression of miRNAs directs development and differentiation of specific cells and tissues, which becomes more complex as the embryo continues to develop (Fig. 1). If disruptions occur to the network of regulatory connections between miRNAs and developmental signalling pathways, then this may lead to incorrect maintenance of signalling, promoting an environment where proliferation is enhanced, and cells are able to evade apoptosis, or remain in a more pluripotent state. In this instance the normal growth regulatory processes have lost control, or are mis-timed, and therefore may initiate tumour growth.

In the childhood tumour types discussed above, there are miRNAs (or clusters) that are clearly implicated in more than one type of tumour (Tables 1, 2 and 3). Identifying key miRNAs, which act as tumour suppressors, or as oncomiRs, is already a growing area of interest, but the discovery of a common miRNA regulated mechanism throughout all childhood solid tumours would be immensely valuable in identifying new targeted therapies [125]. The *miR-17-92* cluster is implicated in several childhood solid tumours and is highlighted in this review in NB, WT, HB and MB; it is a well-researched topic and is known to be vital in early development [126]. Members of the cluster have been found to be ubiquitously expressed in all tissues (except the heart and blood vessels) in early chick embryos, which would suggest these miRNAs play a role in regulating baseline processes that are critical in many different cell and tissue types. The shared expression of this group of miRNAs is an interesting phenomenon and could potentially hold promising targets for the development of new therapies in treating childhood solid tumours [127]. Another set of miRNAs worth further investigation, which are implicated in several paediatric tumours, is the C19MC group (Table 2). The miRNAs in this cluster are also thought to be oncogenic, and appear to be closely linked to early neural tube development, and may act in promoting transformation and maintenance of these early proliferative cell types. The C19MC group appears to be especially critical in the formation of embryonal brain tumours, particularly those with aggressive histology. It would be worth determining if this cluster is active in other childhood solid tumours, or if it is brain specific.

It is critical to uncover the mechanisms by which aberrant developmental signalling occurs and how these processes may be manipulated to regress into a more regulated path without causing additional disruption in embryonic development. Since these events may occur very early, it has thus far been challenging to study miRNA expression in embryo preimplantation. However, miRNA expression in the stages following implantation, at the beginning of embryonic development, is vital and has been widely researched. The discovery of miRNAs themselves were uncovered in the investigation of developmental timing in *C. elegans* [1].

While it is hopeful that elucidation of how miRNAs contribute to the formation of solid tumours can help revolutionise the approach to treating patients, it adds yet another layer of complexity to an already entangled web of interactions occurring both at the genetic and epigenetic level. Currently there are over 2,500 mature human miRNA products identified in humans [128]. Given that each miRNA is able to target tens or even hundreds of different genes, the development of targeted therapies will be difficult, and due to the nature of miRNAs and their ability to be “flexible” in base pair complementarity to their targets, it seems that if specific miRNAs were targeted therapeutically, it might give rise to a compensatory effect from other related miRNAs [129]. In addition, often members of the same family of miRNAs tend to target shared transcripts; this is due to the similarities in the seed region. At the current time communities of miRNAs are still being discovered that share a significant proportion of target genes through co-expression analyses, and therefore there is still much to be uncovered about the integrated functional categories of different miRNAs and how they interact [130]. Ideally, uncovering the initiating events in miRNA expression, (when the first miRNAs are expressed in development and how this takes place), would help inform on how disruptions may occur and allow researchers to strive toward better classification of the contributions of miRNAs in embryonal solid tumours. However, it remains that the number of investigations in the different types of childhood solid tumours are still relatively few; translation of this research into miRNAs, or their upstream counterparts is poised to expand to other solid tumours. This will assist in uncovering highly valuable links between different kinds of tumours. In the future these discoveries may form the basis to develop a type of shared network of miRNAs or specific mutations in biogenesis pathway components that could help in the customisation of current therapeutic treatments, or even the generation of new, targeted therapies.

## Abbreviations

BMP: bone morphogenetic protein
BWS: Beckwith – Wiedemann syndrome
C19MC: Chromosome 19 microRNA cluster
CNS-PNET: Central nervous system primitive neuro-ectodermal brain tumour
COO: Cell of origin
CSC: Cancer stem cell
ETMR: Embryonal tumours with multi-layered rosettes
FAP: Familial adenomatous polyposis
HB: Heptaoblastoma
JAK/STAT: Janus-activated kinase/signal transducer and activator of transcription
KRT19: Keratin 19
MAPK: Mitogen-activated protein kinase
MB: Medulloblastoma
miRISC: Microrna induced silencing complex
miRNA: Micrornas
MRE: Microrna recognition elements
NB: Neuroblastoma
oncomiR: Oncogenic microRNAs
OS: Osteosarcoma
PA: Pediatric pilocystic astrocytoma
PI3K: Phosphatidylinositol
PLAG1: Pleomorphic adenoma gene
TGF: Transforming growth factor
TTYH1: Tweety family member 1
WT: Wilms Tumour
